# Supplementary figures and images for: The phosphoproteomic landscape of the neurological manifestations in tuberous sclerosis complex
Source: Acta Neuropathol. 2026 May 20;151(1):60. doi: 10.1007/s00401-026-03022-5 (PMC13190377; doi:10.1007/s00401-026-03022-5)

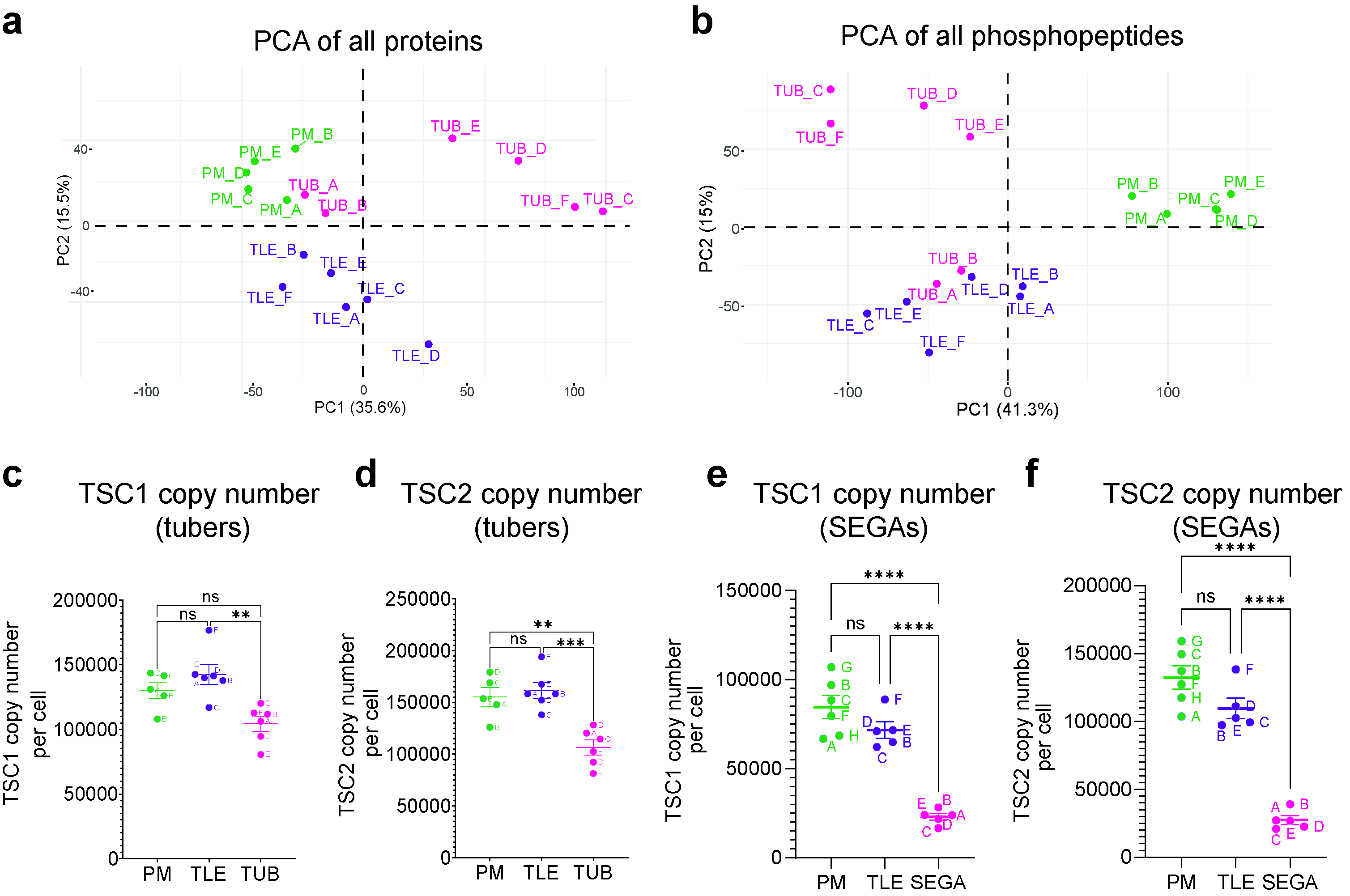

Supplement: Supplementary file 7 — Supplementary file7 (TIF 1635 KB) [file 401_2026_3022_MOESM7_ESM.tif]

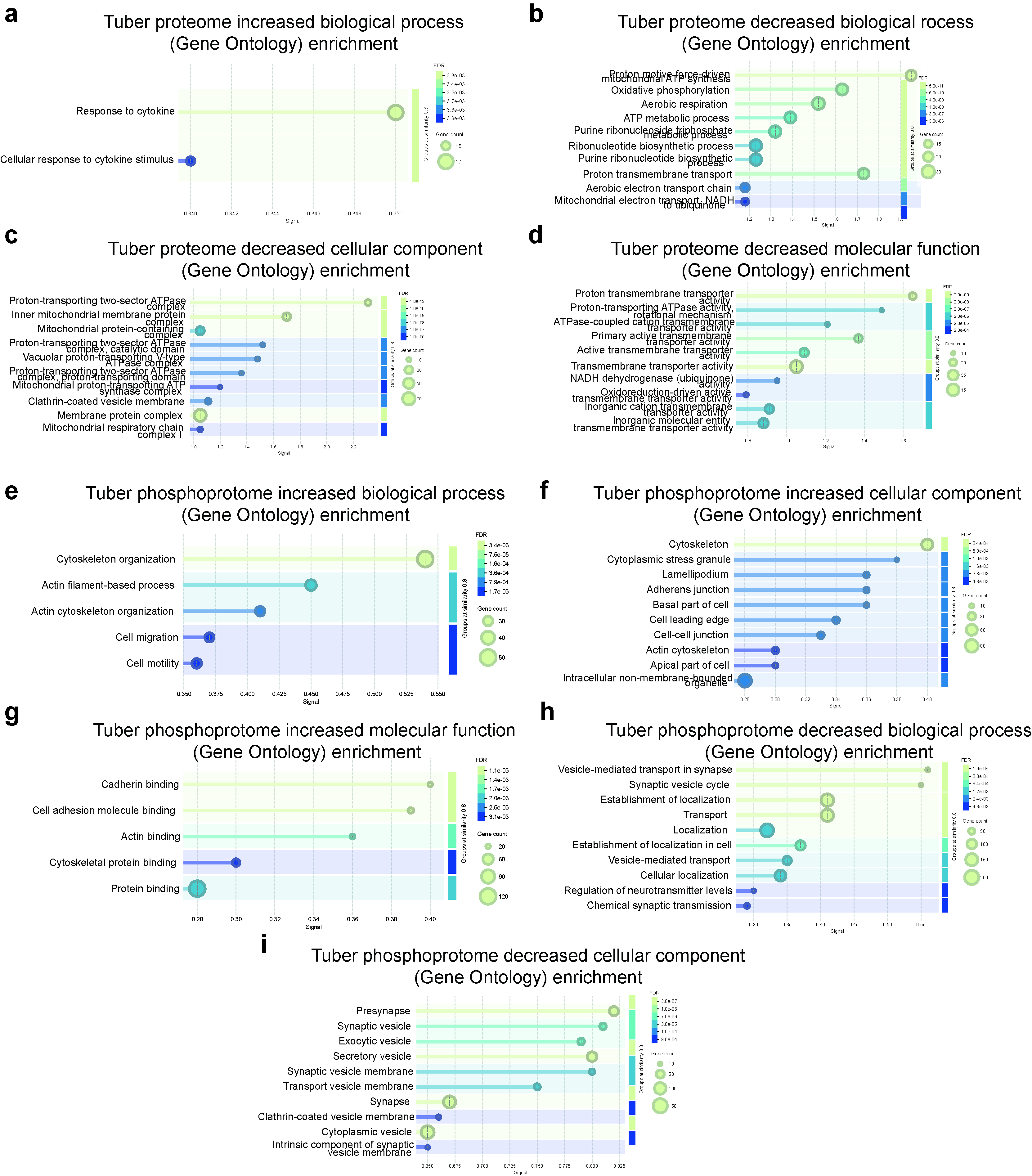

Supplement: Supplementary file 8 — Supplementary file8 (TIF 4282 KB) [file 401_2026_3022_MOESM8_ESM.tif]

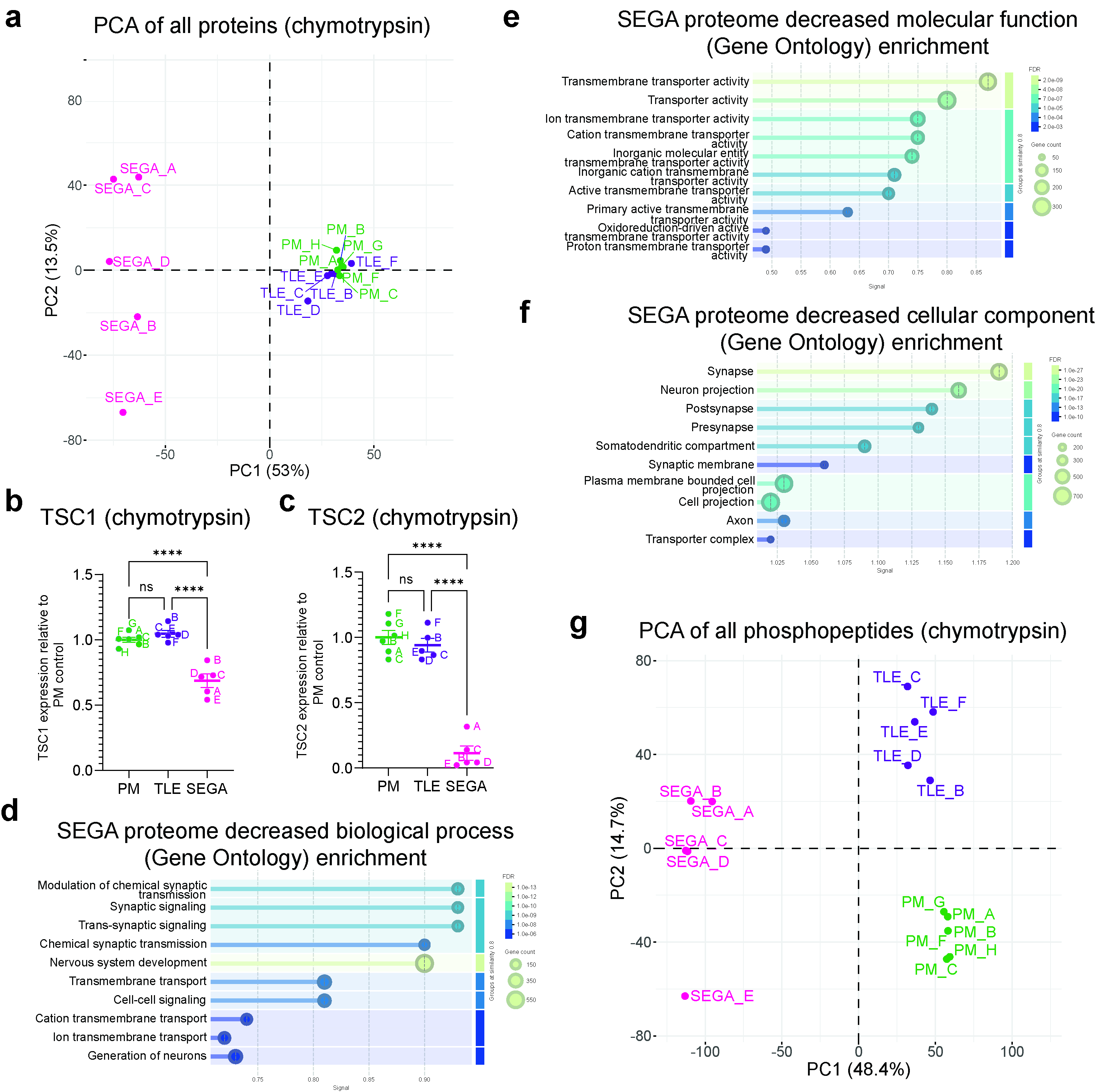

Supplement: Supplementary file 9 — Supplementary file9 (TIF 2968 KB) [file 401_2026_3022_MOESM9_ESM.tif]
