# Supplementary material for: The phosphoproteomic landscape of the neurological manifestations in tuberous sclerosis complex
Source: Acta Neuropathol. 2026 May 20;151(1):60. doi: 10.1007/s00401-026-03022-5 (PMC13190377; doi:10.1007/s00401-026-03022-5)
Supplement: Supplementary file 10 — Supplementary file10 (DOCX 20 KB) [file 401_2026_3022_MOESM10_ESM.docx]

**Supplemental Figure legends**

*Figure S1. Quantification of TSC1 and TSC2 protein copy numbers per cell.* **a** PCA plot of postmortem (PM), temporal lobe epilepsy (TLE) and tuber (TUB) proteomic data. **b** PCA plot of PM, TLE and TUB phosphoproteomic data. **c, d** Copy numbers of TSC1 (**c**) and TSC2 (**d**) proteins in tubers and control tissue. n=5 for PM, n=6 for TLE, n=6 for TUB. **e, f** Copy numbers of TSC1 (**e**) and TSC2 (**f**) proteins in SEGAs and control tissue (trypsin). n=6 for PM, n=5 for TLE, n=5 for SEGA. Data are represented as mean ± SEM and were analysed using one-way ANOVA. ns not significant, * p < 0.05, **p < 0.01, ***p < 0.001****p < 0.0001.

*Figure S2. GO analysis of proteins with altered expression in tubers*. **a** GO analysis of biological process of proteins with increased expression in tubers shows enrichment for cytokines. **b-d** GO analysis of biological process (**b**), cellular component (**c**) and molecular function (**d**) of proteins with decreased expression in tubers shows enrichment for mitochondria-related processes. **e-g** GO analysis of biological process (**e**), cellular component (**f**) and molecular function (**g**) of proteins represented by phosphopeptides with significantly increased expression in tubers shows enrichment for cytoskeleton-related processes. **h, i** GO analysis of biological process (**h**) and cellular component (**i**) of proteins represented by phosphopeptides with significantly decreased expression in tubers shows enrichment for synapse-related processes.

*Figure S3. GO analysis of proteins with decreased expression in SEGAs*. **a** PCA plot of postmortem (PM), temporal lobe epilepsy (TLE) and SEGA proteomic data in the chymotrypsin condition. **b, c** TSC1 (**b**) and TSC2 (**c**) protein expression levels are strongly decreased in the chymotrypsin condition. **d-f** GO analysis of biological process (**d**), molecular function (**e**) and cellular component (**f**) of proteins with significantly decreased expression in SEGAs shows enrichment for synapse-related processes. **g** PCA plot of PM, TLE and SEGA phosphoproteomic data in the chymotrypsin condition.

**Supplemental Tables**

*Table S1. Proteomics data for the tuber experiment.*

*Table S2. Phosphoproteomics data for the tuber experiment.*

*Table S3. Direct mTORC1 substrate phosphosites detected in tuber phosphoproteomics.*

*Table S4. Proteomics data for the SEGA experiment (trypsin).*

*Table S5. Proteomics data for the SEGA experiment (chymotrypsin).*

*Table S6. Phosphoproteomics data for the SEGA experiment (trypsin).*

*Table S7. Phosphoproteomics data for the SEGA experiment (chymotrypsin).*

*Table S8. Direct mTORC1 substrate phosphosites detected in SEGA phosphoproteomics.*

*Table S9. SEGA phosphopeptides with significantly increased expression compared to PM or TLE controls (p<0.005, FC ≥ 1) in trypsin and chymotrypsin experiments.*

*Table S10. SEGA proteins represented by phosphopeptides with significantly increased expression compared to PM or TLE controls in trypsin and chymotrypsin experiments.*

*Table S11. Genes with mis-regulated splicing in SEGA tissue identified by at least two analysis pipelines.*

*Table S12. Genes with mis-regulated splicing in SEGA tissue identified by all three analysis pipelines.*

*Table S13. Comparison of CASE dataset with genes mis-regulated splicing genes in SEGAs.*
